# Multiple Oral Leiomyomas in an Infant: A Rare Case

**DOI:** 10.1155/2012/804305

**Published:** 2012-06-03

**Authors:** Efraín Alvarez, María P. Laberry, Carlos M. Ardila

**Affiliations:** ^1^Department of Oral and Maxillofacial Surgery, School of Dentistry, University of Antioquia, Calle 64 No. 52–59, Medellín, Colombia; ^2^Department of Maxillofacial Surgery, University of Antioquia, Calle 64 No. 52–59, Medellín, Colombia; ^3^Biomedica Stomatology Group, School of Dentistry, University of Antioquia, Calle 64 No. 52–59, Medellín, Colombia

## Abstract

Oral leiomyoma is a benign smooth muscle tumor that occurs most frequently in the uterine myometrium, gastrointestinal tract, and skin. Incidence in the oral cavity is considered uncommon. Most cases are reported in adults, with very few cases described in children. A rare case of multiple leiomyomas localized on the tongue, cheek, and floor of the mouth of an 8-month-old baby is reported. The diagnosis of leiomyoma in the oral cavity is mainly determined by histological studies; however, immunohistochemical tests are recommended in order to differentiate from other tumors. Surgical excision of the lesion appears to be the best treatment option. A review of the literature did not reveal any previously reported case of multiple oral leiomyomas.

## 1. Introduction

Leiomyomas are benign mesenchymal tumors arising from smooth muscles. They frequently occur in the uterus and gastrointestinal tract, but they can initiate wherever smooth muscle cells subsist. Histologically, there are three types of leiomyomas: leiomyoma (solid), angiomyoma (vascular leiomyoma), and the rare form of epithelioid leiomyoma (leioblastoma). Oral leiomyoma is a rare tumor, with most of the cases noted in adults and few cases reported in children. The first case of a mandibular intraosseous leiomyoma in an 8-month-old baby was recorded by Bertolini et al. [1]. The tumor generally manifests as a slow-growing painless lesion, often of a purplish color. Since leiomyomas cannot be clinically distinguished from fibromas, the diagnosis is exclusively based on the histological findings. In order to accomplish more detailed analysis and more exact differential diagnosis, immunohistochemical studies are recommended. A case of multiple leiomyomas localized on the tongue, cheek, and floor of the mouth with their histologic and immunohistochemical profile is presented. A review of the literature did not reveal any previously reported case of multiple oral leiomyomas.

## 2. Case Report

**Figure 1:**
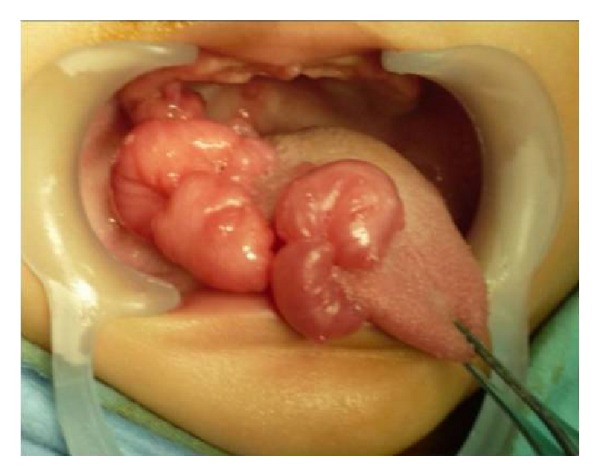
Clinical characteristics of the vascular leiomyomas. Multiple erythematous lesions on the tongue, cheek, and floor of the mouth were observed.

**Figure 2:**
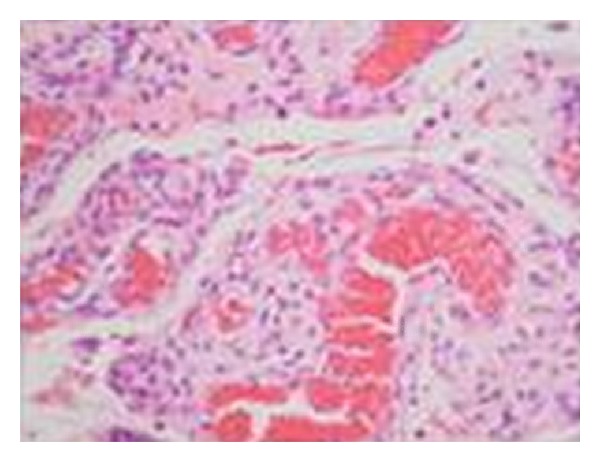
Histopathological characteristics of the vascular leiomyomas. Microscopically fusiform cell proliferation with elongated nuclei and eosinophilic cytoplasm was observed.

**Figure 3:**
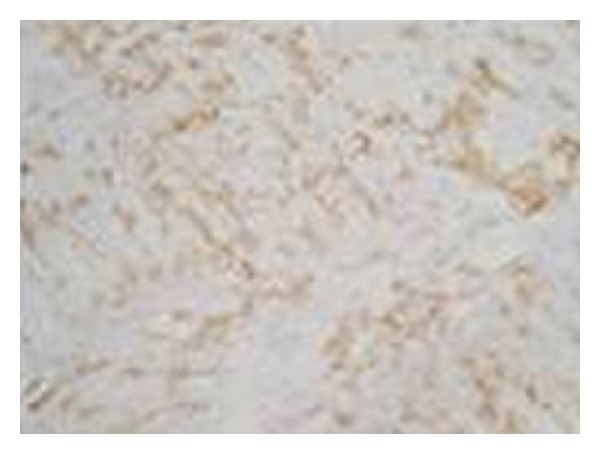
Immunohistochemical characteristics of the vascular leiomyomas. The immunohistochemical study revealed the expression of vimentin, desmin, muscle specific actin, and smooth muscle actin within the tumor cells.

**Figure 4:**
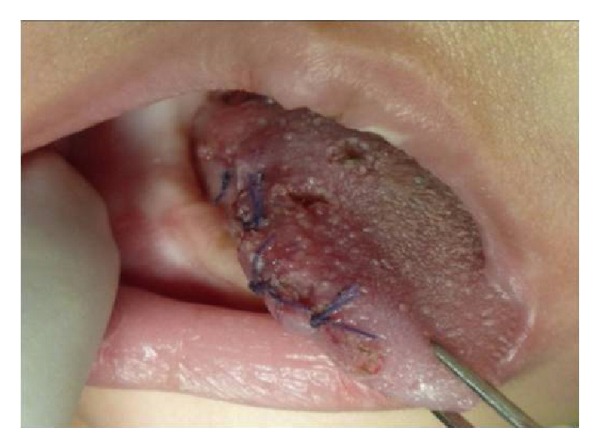
Resection of the leiomyomas. The patient had a correct evolution without any postoperative incident.

An 8-month-old girl was referred to the Department of Oral and Maxillofacial Surgery of the University Hospital San Vicente in Medellín, Colombia, for evaluation of multiple erythematous lesions on the tongue, cheek and floor of the mouth (Figure 1). The lesions had been present for the previous four months. Physical examination revealed well-demarcated nodules measuring around 1 cm in diameter. The overlying mucosa appeared normal and nonulcerated. In order to complete a histological analysis, excisional biopsies were performed. The surgical samples were fixed in 10% buffer formalin for a minimum of 48 hours, embedded in paraffin, and cut at 5 *μ*m to be stained with Hematoxylin-Eosin technique. Microscopically, fusiform cell proliferation with elongated nuclei and eosinophilic cytoplasm was observed. Several blood vessels lined by a thin layer of endothelial cells were observed intercalated in the fascicules (Figure 2). Immunohistochemical techniques were also applied, involving monoclonal antibodies against actin, vimentin, cytokeratin, and the S-100 protein (Figure 3). The immunohistochemical study revealed the expression of vimentin, desmin, muscle specific actin (MSA), and smooth muscle actin (SMA) within the tumor cells. The other markers studied (cytokeratin, AE1/AE3, EMA, S100, and CD34) were negative. Diagnosis of vascular leiomyomas was confirmed. Computerized tomography ruled out bone association, corroborating the completely mucosal allocation of the leiomyomas. Finally, the lesions were removed under general anaesthesia (Figure 4). The patient had a correct evolution without any postoperative incident. The patient returned after a 6-month period, at which time there was no evidence of recurrence.

## 3. Discussion

To the best of our knowledge, this is the first case of multiple leiomyomas in an infant. Oral leiomyomas are considered uncommon neoplasms, representing only 0.016% to 0.065% of all the leiomyomas [2]. The vascular variant is the most frequent in the oral cavity with 75% of all cases corresponding to this histological type [2–4]. The greater occurrence of this variation is related to the most frequent source of smooth muscle in the oral cavity, represented by the wall of blood vessels [5]. The highest prevalence of head and neck leiomyoma is observed between 40 and 49 years of age [2–4] and the lips are the most common site followed by the tongue, cheeks, palate, and gingivae [3]. In the present case, the patient was 8 months old at the moment of her diagnosis and several leiomyomas were localized in the floor of the mouth, representing an unusual case. Similarly, Bertolini et al. [1] reported a leiomyoma in an 8-month-old, but the diagnosis was a mandibular intraosseous lesion. The gender preference for females is in agreement with the literature [1–5].

Most of the vascular leiomyomas are nodular, painless, slow growth lesions, less than 2 cm in diameter, and of a color that can vary from white to blue [6]. This case corroborates with what has been published previously. From the clinical appearance, it is very difficult to differentiate a leiomyoma from other mesenchymal tumors such as fibromas, neurofibromas, lipomas, mucoceles or the leiomyosarcoma, the malignant counterpart [1–5, 7], and therefore the final diagnosis of oral leiomyoma is mainly determined by a histological and immunohistochemical study.

The vascular leiomyoma is characterized by a well-defined proliferation of mesenchymal tapered cells with eosinophilic cytoplasm and elongated basophilic nuclei that show tapered endings [4]. Immunohistochemically, leiomyomas are reactive with vimentin, desmin, *α*-smooth muscle actin, and muscle specific actin. In this context, the immunohistochemical technique is an important aid, in which these tumors express immunoreactivity for SMA and negativity for the S-100 protein. In this case, fusiform and epithelial-like cell areas were observed and the immunohistochemical study revealed the expression of vimentin, desmin, MSA, and SMA within the tumour cells. Immunohistochemical findings similar to those noted in the case presented here have also been reported [4, 6].

Wide surgical resection is the most reported treatment in reviewed literature with successful results. However, to remove the tumor, a carbon dioxide laser has been used [7]. Recurrence rate is very low if complete resection is achieved [8].
